# Frailty and long-term survival of patients with gastric cancer: a meta-analysis

**DOI:** 10.3389/fonc.2023.1239781

**Published:** 2023-09-21

**Authors:** Hongliang Liang, Aiping Hu

**Affiliations:** ^1^ Department of Gastroenterology, Liaocheng People’s Hospital, Liaocheng, China; ^2^ Department of Oncology, Liaocheng Tumor Hospital, Liaocheng, China

**Keywords:** gastric cancer, frailty, recurrence, survival, meta-analysis

## Abstract

**Background:**

The relationship between frailty and the long-term clinical outcome of gastric cancer (GC) patients has not yet been established, although frailty is associated with a poor short-term outcome. The impact of frailty on long-term survival of GC patients was investigated through a systematic review and meta-analysis.

**Methods:**

Observational studies with longitudinal follow-ups for a minimum of one year were identified through a search of the PubMed, Embase, Cochrane Library, and Web of Science databases, in accordance with the objective of the meta-analysis. Combining the findings was achieved using a random-effects model, which accounted for inter-study heterogeneity.

**Results:**

Ten datasets from nine cohort studies were included, which involved 7613 patients with GC. A total of 2074 patients (27.2%) were with frailty at baseline, and the mean follow-up duration was 48.1 months. A pooled analysis of the results showed that frailty was linked to a poor long-term overall survival in GC patients (risk ratio [RR]: 1.65, 95% confidence interval [CI]: 1.27 to 2.13, p < 0.001; I^2^ = 80%). Sensitivity analysis showed consistent results in older patients (≥ 65 years, RR: 1.51, p = 0.002) and the oldest old (≥ 80 years, RR: 1.41, p = 0.01). In addition, frailty was also associated with poor long-term progression-free survival (RR: 1.65, 95% CI: 1.39 to 1.96, p < 0.001; I^2^ = 0%) and disease-specific survival (RR: 1.71, 95% CI: 1.23 to 2.37, p = 0.001; I^2^ = 4%).

**Conclusion:**

Frailty is associated with poor long-term survival of patients with GC.

## Introduction

More than 1 million gastric cancers are diagnosed every year around the world, making it the fifth most common cancer worldwide (1, 2). The current treatments for GC involve surgical resection, radiotherapy, chemotherapy, target treatment, and immunotherapies, depending on the histopathological characteristics of the cancer and the functional status of the patients (3, 4). For individual patient with GC, despite of the multiple therapeutic modalities, the survival could vary significantly (5). Thus, it is necessary to determine the risk factors linked to poor prognoses in patients suffering from GC. In geriatrics, frailty is characterized by diminished physical capacity and functioning across multiple organ systems (6, 7). There is increasing evidence that frailty is associated with poor clinical outcomes in patients with various clinical conditions, such as cancer (8). In oncology, comprehensive geriatric assessments (CGAs) have increasingly been used to evaluate frailty as a risk factor for malignancy in older populations (9). For patients with GC receiving gastrectomy, a meta-analysis in 2022 showed that there is a high risk of poor short-term survival and a high rate of readmission within one year in those with frailty (10). However, the long-term influence of frailty on survival of GC, in patients who were treated both surgically and non-surgically, remains not fully determined. In viewing the inconsistent results of previous studies (11–19), our objective was to examine the influence of frailty on long-term prognosis of patients with GC through a systematic review and meta-analysis.

## Methods

Based on MOOSE guidelines (20)and PRISMA statement (21), the meta-analysis was designed, conducted, and reported.

### Literature search

By combining the following terms, PubMed, Embase, Cochrane Library, and Web of Science were systematically searched: (1) “frailty” OR “frail”; (2) “gastric” OR “stomach”; and (3) “cancer” OR “tumor” OR “carcinoma” OR “neoplasm” OR “adenocarcinoma” OR “malignancy”. The scope of the inquiry was restricted to English-language human studies, with additional scrutiny of the bibliographies of both primary and secondary sources. The ultimate exploration of the literature was conducted on March 15, 2023.

### Study selection

A “PICOS” principle was followed in designing the inclusion criteria.

P (patients): patents with clinically diagnosed GC, with no restrictions of clinical stage or treatment;
I (exposure): patients with frailty at baseline; the details of frailty diagnosis diagnostic were in accordance t with the methods described among the included studies;
C (control): patients without frailty at baseline;
O (outcome): reported at least one of the following outcomes between GC patients with and without frailty during follow-up for at least one year, such as overall survival (OS), progression-free survival (PFS), and disease-specific survival (DSS) of GC.
S (study design): Research with longitudinal follow-up that include cohorts, case-control pairs, and post-hoc analyses of clinical trials, published as full-length articles in English.

The meta-analysis excluded reviews, editorials, cross-sectional studies, and studies not relevant to the goal, as well as studies that were not relevant to the goal.

### Data collection and quality evaluation

According to predefined inclusion criteria, two authors independently searched for literature, extracted data, and assessed study quality. Whenever disagreements were found, the two authors discussed these inconsistencies until consensus was reached. Variables regarding study information, patient characteristics, methods of frailty assessment, follow-up duration, and outcome data were collected. Using the Newcastle-Ottawa Scale (NOS) (22), nine stars were assigned to each study for quality assessment based on three aspects: choosing study groups, between- group comparability, and outcome analysis.

### Statistical analyses

Risk ratios (RRs) corresponding 95% confidence interval (CI) were used as the variables to indicate the association between frailty and long-term survival of patients with GC. A logarithmical transformation was performed on the RR and its corresponding stand error (SE) from each study to stabilize and normalize its variance (23). We conducted the Cochrane Q test to evaluate the heterogeneity of the included studies (23, 24), as well as the calculation of I^2^ statistic. If I^2^ > 50%, a significant heterogeneity was considered. By taking into account potential heterogeneity among the included studies, we pooled the results using a random-effects model. Sensitivity analysis was performed to evaluate the age of the patients on the outcome by limiting the analyses to studies including older patients (≥ 65 years) and the oldest old (≥ 80 years) only. In order to evaluate the impact of predefined study characteristics on results, predefined subgroup analyses were performed according to study design, prevalence of frailty, follow-up duration, and study quality scores. By inspecting funnel plot symmetry and performing Egger regression tests, potential publication bias was assessed (25). The RevMan (Version 5.1; Cochrane Collaboration, Oxford, UK) and Stata (version 12.0; Stata Corporation, College Station, TX) software were used for the statistics.

## Results

### Literature search

**Figure 1:**
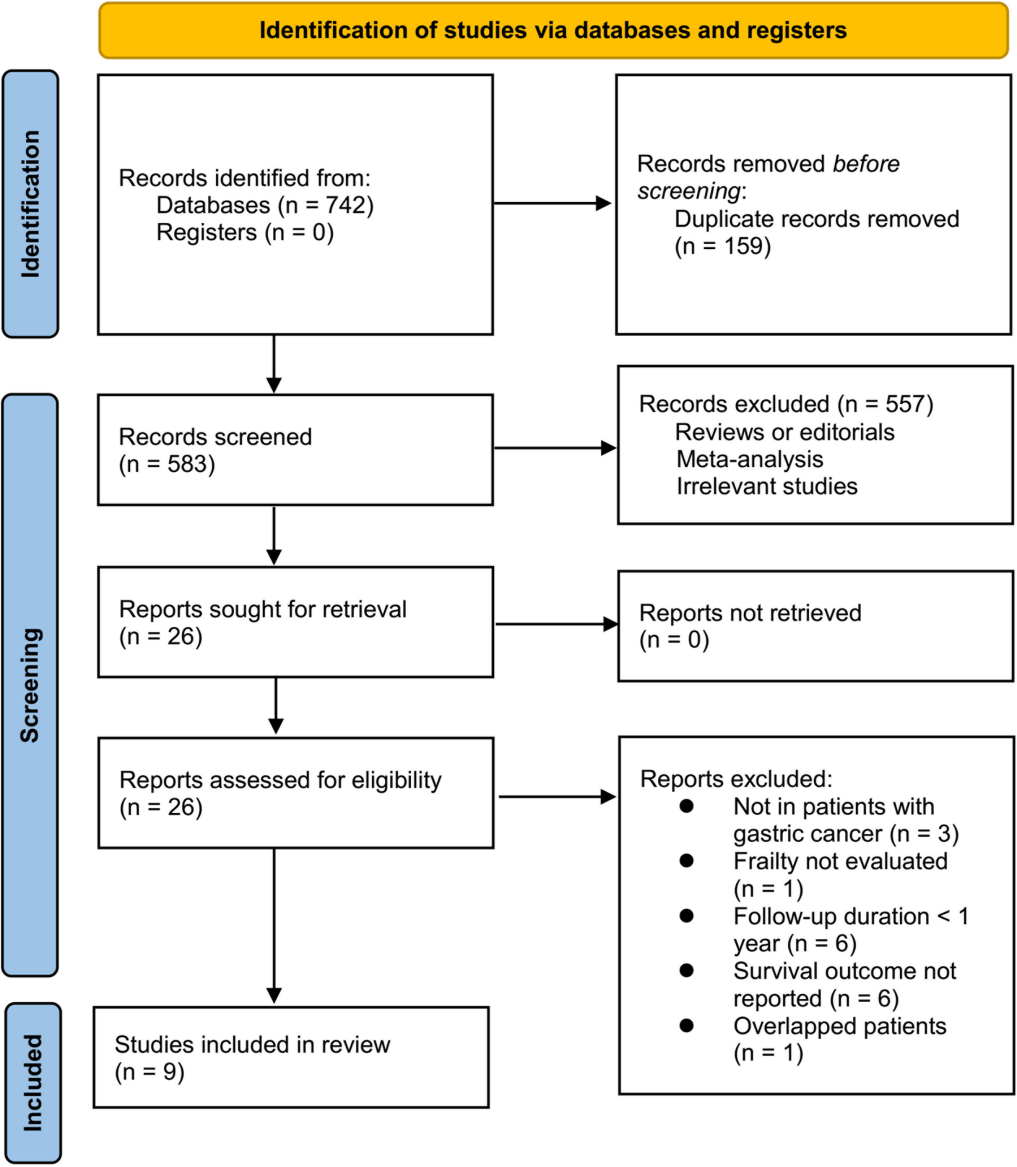
Flowchart of database search and study identification.

Briefly, 742 studies were enrolled in the primary database search, and 159 were excluded due to the duplications. In the remaining 583 studies, 557 were excluded mainly because the relevancy was lacking. Seventeen of the remaining 26 studies that were subjected to full-paper reading were further excluded due to the reasons listed in **Figure 1**. This meta-analysis finally incorporated nine studies (11–19).

### Study features and quality

Overall, nine cohort studies, three prospective (11, 12, 15) and six retrospective (13, 14, 16–19), were enrolled for analysis (**Table 1**). These studies were performed in Japan, Korea, China, and United Kingdom, and published within 2017~2023. A total of 7613 patients with GC were involved in these studies. As for the clinical cancer stage, six studies included patients with stage I-III GC (11–16), while the remaining three included patients with stage I-IV GC (17–19). The main treatment for the patients were surgical resection in six studies (11–13, 15–17), endoscopic submucosal dissection in one study (14), chemotherapy in one study (18), and a comprehensive treatment with surgery, radiotherapy, and chemotherapy in another study (19). Two studies included adult GC patients (16, 18), while the other seven studies included older patients with GC (15, 17) and the oldest old with GC (11–14, 19), respectively. Different frailty evaluating scales were used among these studies, such as the chart-derived frailty score (11, 12), the Clinical Frailty Scale (13, 14) and the modified Clinical Frailty Scale (18), the multidimensional frailty score (15), the hospital frailty risk score (16), the Study of Osteoporotic Fractures scale (17), and the multidimensional frailty score (19). Accordingly, a total of 2074 patients (27.2%) were frail at baseline. The studies included in this analysis had follow-up durations ranging from 12 to 54 months, with a mean duration of 48.1 months. In all studies, confounding factors such as age, sex, tumor stage, and comorbidities were adjusted to varying degrees. A NOS score ranging from 7 to 9 indicates that the included follow-up studies were generally of high quality (see **Table 2**).

**Table 1:** Characteristics of the included studies.

Study	Country	Design	Tumor stage	Main treatment	Patient number	Age	Male (%)	Frailty evaluation	Number of patients with frailty	Median follow-up duration (months)	Outcomes reported	Variables adjusted
Lu 2017	China	PC	I-III	Radical open gastrectomy	165	> 80 years, median 81.4 years	80	CDFS	54	31	OS, DSS, PFS	Age, sex, tumor stage, location, size, histological type, and ASA score
Lu 2018	China	PC	I-III	Radical laparoscopic gastrectomy	119	> 80 years	81.5	CDFS	43	37	OS, DSS, PFS	Age, sex, ASA class, BMI, comorbidities, and adjuvant chemotherapy
Tanaka 2019	Japan	RC	I-III	Radical laparoscopic gastrectomy	96	80 years or older	72.9	CFS	17	40	OS, DSS	Age, sex, tumor stage, BMI, PS, ASA class, CCI, and HGB
Misawa 2020	Japan	RC	I-III	Endoscopic submucosal dissection	142	80 years or older	64.1	CFS	41	48	OS	Age, sex, BMI, CCI, ASA score, and PNI
Kim 2021	Korea	PC	I-III	Radical gastrectomy	289	66~94 years	63.3	MFS	111	12	OS	Age, sex, tumor stage, ASA score, type of surgery, handgrip strength, and walk speed
Kouzu 2021	Japan	RC	I-III	Radical gastrectomy	430	Mean: 69.3 years	75.1	FRAS	92	48	OS, PFS	Age, sex, BMI, CCI, tumor size, depth, stage, histological type, and CONUT score
Pearce 2022	UK	RC	I-IV	Chemotherapy	514	51~96 years, median: 76 years	74.9	Modified CFS	230	13	OS, PFS	Age, sex, metastases, planned use of trastuzumab, and dose of chemotherapy
Jeong 2022	Korea	RC	I-IV	Gastrectomy	231	65 years or older, median: 72 years	60.6	SOF index	35	48	OS	Age, sex, BMI, PS, CCI, ASA Score, tumor histological type, stage, and adjuvant chemotherapy
Zhang 2023	Korea	RC	I-IV	Surgery, radiotherapy, chemotherapy,	5627	85 years or older	44.8	HFRS	1451	54	OS	Age, sex, CCI, income, and treatment

PC, prospective cohort; RC, retrospective cohort; CDFS, chart-derived frailty score; CFS, Clinical Frailty Scale; SOF, Study of Osteoporotic Fractures; MFS, multidimensional frailty score; FRAS, Fall Risk Assessment Score; HFRS, hospital frailty risk score; OS, overall survival; PFS, progression-free survival; DSS, disease-specific survival; ASA, American Society of Anesthesiologists; BMI, body mass index; CCI, Charlson Comorbidity Index; PS, physical status; HGB, hemoglobin; PNI, prognostic nutritional index; CONUT; Controlling Nutritional Status;

**Table 2:** Quality evaluation of the included studies.

Study	Representativeness of the exposed cohort	Selection of the non-exposed cohort	Ascertainment of exposure	Outcome not present at baseline	Control for age and sex	Control for other confounding factors	Assessment of outcome	Enough long follow-up duration	Adequacy of follow-up of cohorts	Total
Lu 2017	1	1	1	1	1	1	1	1	1	9
Lu 2018	0	1	1	1	1	0	1	1	1	7
Tanaka 2019	1	1	1	1	1	1	1	1	1	9
Misawa 2020	0	1	1	1	1	0	1	1	1	7
Kim 2021	0	1	1	1	1	1	1	1	1	8
Kouzu 2021	1	1	1	1	1	1	1	1	1	9
Pearce 2022	0	1	1	1	1	1	1	1	1	8
Jeong 2022	0	1	1	1	1	1	1	1	1	8
Zhang 2023	0	1	1	1	1	0	1	1	1	7

### Results of meta-analysis

**Figure 2:**
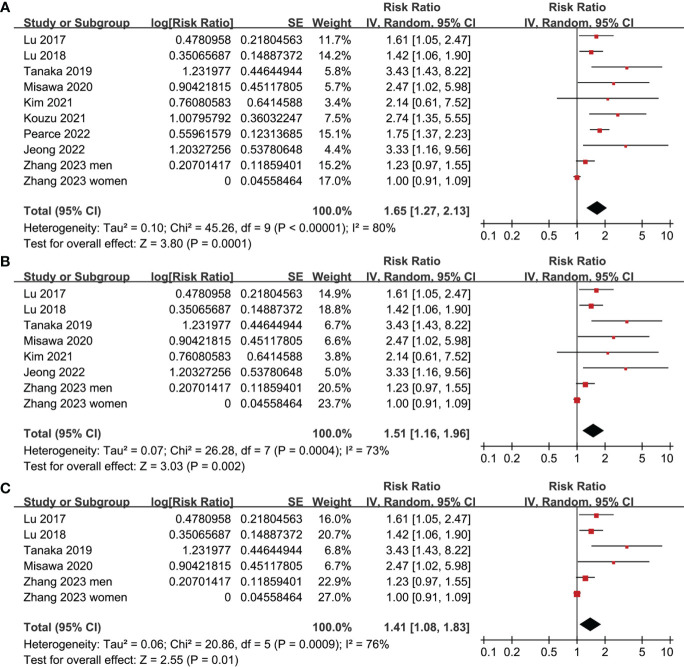
Meta-analysis for the association between frailty and OS of patients with GC; **(A)** meta-analysis of overall population; **(B)** sensitivity analysis in older patients; and **(C)** sensitivity analysis in the oldest old.

**Figure 3:**
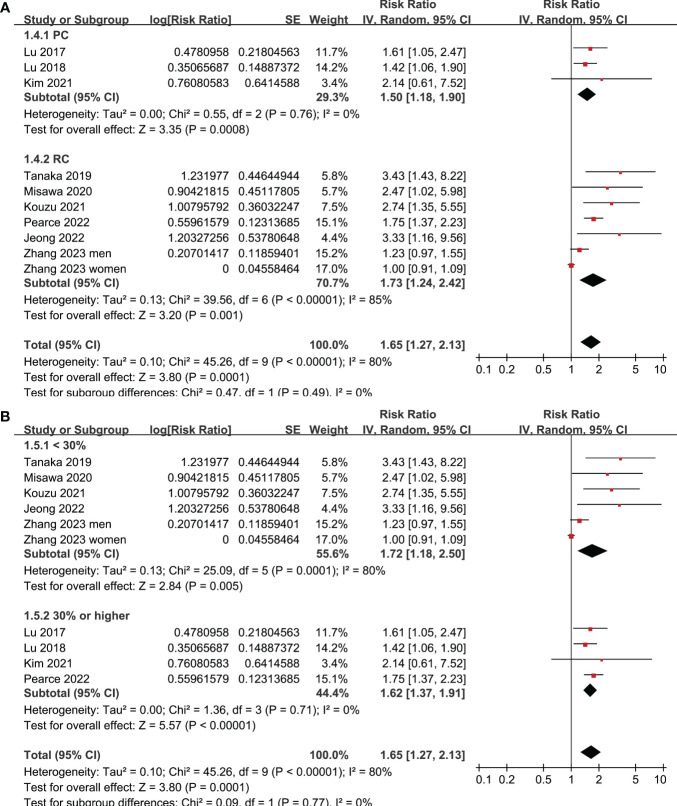
Meta-analysis for the subgroup analysis of the association between frailty and OS of patients with GC; **(A)** subgroup analysis according to study design; and **(B)** subgroup analysis according to the prevalence of frailty.

**Figure 4:**
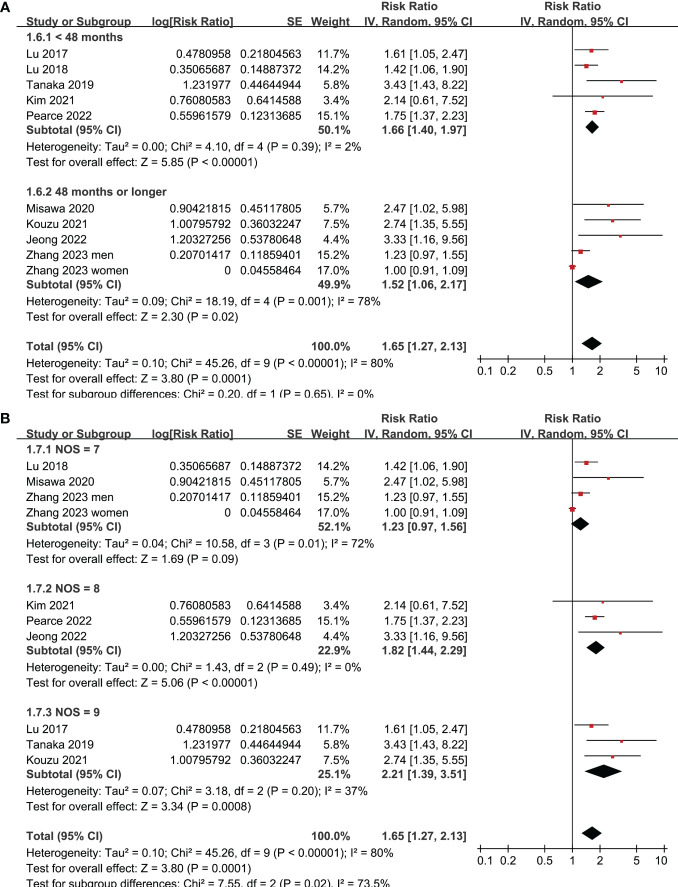
Meta-analysis for the subgroup analysis of the association between frailty and OS of patients with GC; **(A)** subgroup analysis according to follow-up durations; and **(B)** subgroup analysis according to study quality scores.

**Figure 5:**
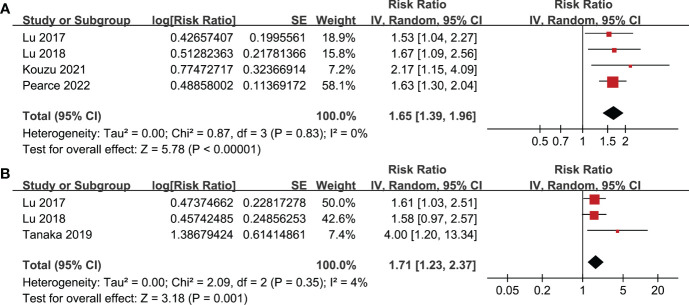
Meta-analysis for the association between frailty and other survival outcomes of patients with GC; **(A)** meta-analysis for the association between frailty and PFS of patients with GC; and **(B)** meta-analysis for the association between frailty and DSS of patients with GC.

All studies incorporated in the analysis reported the outcome of OS. One study (19), however, presented the association between frailty and OS based on the sex of the patients, resulting in the independent inclusion of these datasets in the meta-analysis. The pooled results of ten datasets from nine studies indicated that frailty was significantly associated with unfavorable long-term OS in patients diagnosed with GC (RR: 1.65, 95% CI: 1.27 to 2.13, p < 0.001; I^2^ = 80%; **Figure 2A**). Sensitivity analysis showed consistent results in older patients (≥ 65 years, RR: 1.51, 95% CI: 1.16 to 1.96, p = 0.002; I^2^ = 73%; **Figure 2B**) and the oldest old (≥ 80 years, RR: 1.41, 95% CI: 1.08 to 1.83, p = 0.01; I^2^ = 76%; **Figure 2C**). In addition, subgroup analysis showed that the association between frailty and poor long-term OS of patients with GC were not significantly affected by study design (**Figure 3A**), prevalence of frailty (**Figure 3B**), or mean follow-up durations (**Figure 4A**, p for subgroup differences all > 0.05). While difference of NOS may affect the results, frailty was shown to be associated with poor OS of GC in studies with eight (RR: 1.82, 95% CI: 1.44 to 2.29, p < 0.001; I^2^ = 0%) or nine points on NOS (RR: 2.21, 95% CI: 1.39 to 3.51, p < 0.001; I^2^ = 37%), but not in studies with NOS of seven points (RR: 1.23, 95% CI: 0.97 to 1.56, p = 0.09; I^2^ = 72%; p for subgroup difference = 0.02; **Figure 4B**). In addition, pooled results of four (11, 12, 16, 18) and three (11–13) studies also showed that frailty was associated with poor long-term PFS (RR: 1.65, 95% CI: 1.39 to 1.96, p < 0.001; I^2^ = 0%; **Figure 5A**) and DSS (RR: 1.71, 95% CI: 1.23 to 2.37, p = 0.001; I^2^ = 4%; **Figure 5B**) in patients with GC.

### Publication bias

**Figure 6:**
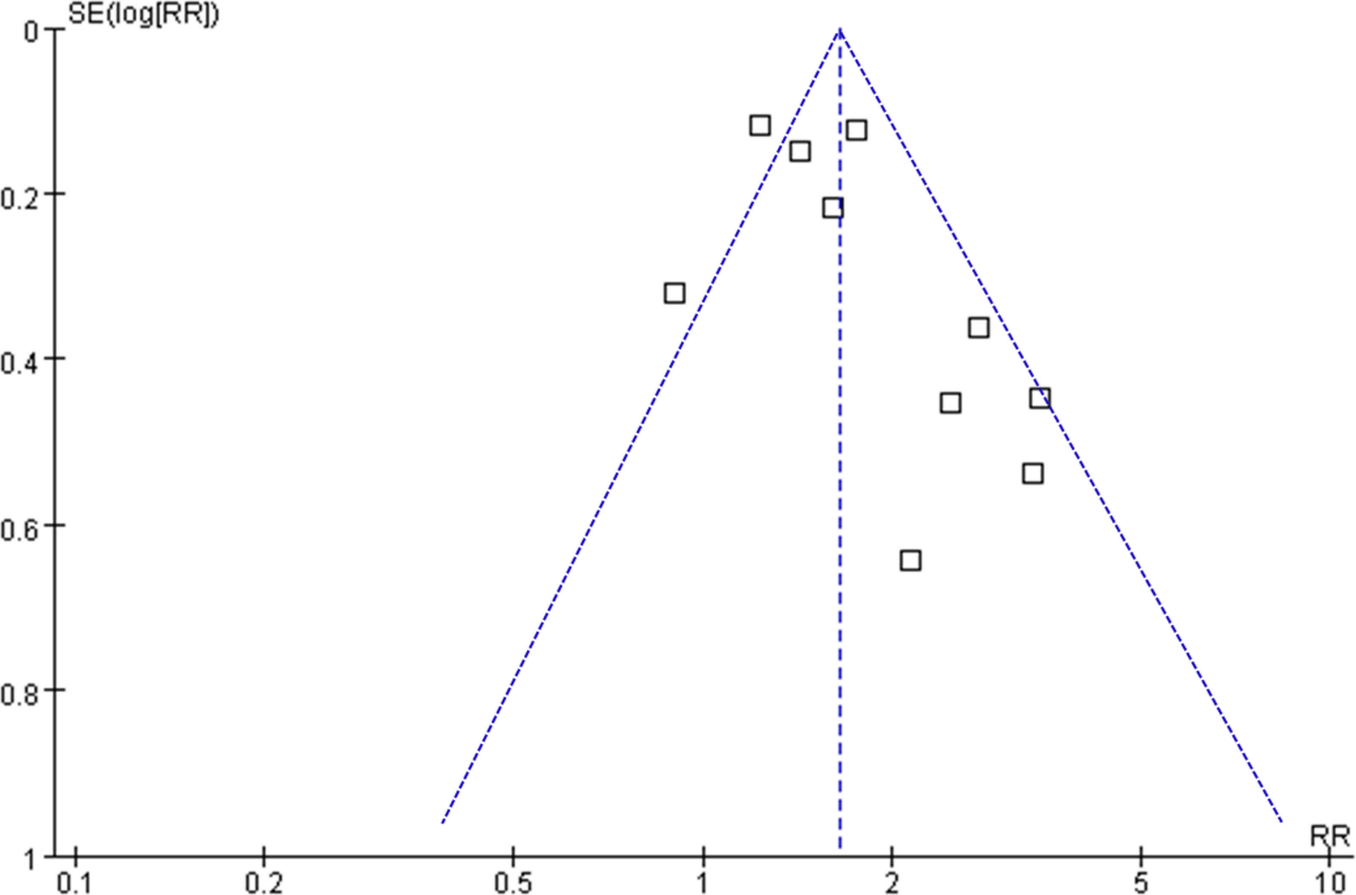
Funnel plots for the meta-analyses of the association between frailty and OS of patients with GC.

**Figure 6** depicts the funnel plots utilized in the meta-analysis of frailty and long-term OS in patients diagnosed with GC. The plots exhibit a symmetrical appearance, indicating a low risk of publication bias. Furthermore, the results of Egger’s regression tests support the notion of low publication biases underlying the meta-analyses (P = 0.22). However, due to the limited number of studies included for PFS and DSS, it was not possible to determine the publication biases underlying the meta-analyses for these outcomes.

## Discussion

The systematic review and meta-analysis findings suggest that patients diagnosed with GC who display frailty at baseline are linked to a lower overall survival rate over a mean follow-up duration of 48.1 months, in contrast to those who do not exhibit frailty. Sensitivity analysis indicates that this association persists among older patients and the oldest old. Additionally, subgroup analysis reveals that the study design, prevalence of frailty at baseline, and follow-up duration do not significantly impact the outcomes. While variations in study quality scores may have a notable impact on outcomes, research indicates that the correlation between frailty and unfavorable OS in patients with GC persists in studies with NOS of eight or nine. Additionally, frailty has been linked to inferior PFS and DSS in GC patients. Combined, these results suggest that frailty may contribute to suboptimal long-term survival outcomes in GC. As far as our current understanding goes, two meta-analyses have been conducted to assess the significance of frailty in patients with GC. In a previous meta-analysis conducted in 2017, Shen et al. examined the impact of frailty on postoperative events in older patients with GC who were undergoing gastrectomy surgery (26). However, only one study meeting the eligibility criteria was identified, which demonstrated that frailty may be related to poor in-hospital survival (26). In a subsequent meta-analysis in 2022, Wang et al. included eight studies of GC patients after gastrectomy, and showed that frailty was associated with a reduced postoperative survival and an increased risk of readmission one year after the surgery (10). Although these results suggested a potential prognostic role of frailty in patients with GC, only patients who were treated surgically were included and only short-term outcomes were observed. The impact of frailty on long-term prognosis of patients with GC remains not fully determined. Compared the previous meta-analyses, current study has several methodological strengths. Initially, we incorporated studies with a follow-up duration of no less than one year and did not impose any limitations on the primary treatment for GC, with the objective of assessing the influence of frailty on the extended outcomes of GC patients. Subsequently, we conducted a comprehensive literature search across four frequently utilized databases, which yielded nine current and pertinent studies. Furthermore, all of the studies included were cohort studies, which could furnish a longitudinal association between frailty and unfavorable clinical outcomes in patients. Moreover, the studies incorporated in this meta-analysis employed multivariate analysis to control for confounding variables, indicating a plausible autonomous correlation between frailty and unfavorable long-term survival outcomes in patients with GC. Additionally, the outcomes of numerous sensitivity and subgroup analyses were consistent, thereby reinforcing the reliability of the results. In summary, our meta-analysis extends the existing literature by demonstrating that frailty may serve as a prognostic factor for poor long-term survival in patients with GC. According to these findings, frailty may contribute to a suboptimal long-term prognosis of patients with GC.

Frailty is likely associated with poor survival among patients with GC due to multiple mechanisms. In GC patients after gastrectomy, frailty was associated with a higher risk of postoperative complications, which may impair long-term survival (27, 28). Additionally, cancer patients with frailty are likely to be less tolerant of effective anticancer treatments, such as surgeries (29) and chemotherapy (30), which may lead to suboptimal therapeutic efficacies. In addition, recent studies showed that frailty may also be associated with a higher risk of toxicity related to chemotherapy (31, 32). Pathophysiologically, frailty has been related with a low-degree systemic inflammation and impaired immunity with cancer, which have been involved in the progression of cancer (33). Although the molecular mechanisms remain to be determined, results of the meta-analysis support a connection between frailty and poor survival of GC patients. Furthermore, studies may be conducted to determine if interventions targeting frailty conditions can improve long-term survival in GC patients.

This study is subject to certain limitations. Firstly, the diagnostic techniques and criteria employed to identify frailty differed among the studies included in the meta-analysis, resulting in heterogeneity that may have impacted the findings. Nonetheless, there is currently no consensus on the most effective screening tool for frailty in cancer patients. Secondly, six of the studies analyzed were retrospective in nature, which could have introduced recall and selective biases. However, subgroup analysis based on study design yielded comparable outcomes. Moreover, despite the implementation of multivariate analyses, the potential for residual confounding factors influencing the correlation between frailty and unfavorable survival outcomes in GC cannot be entirely ruled out. Furthermore, the meta-analysis solely comprised observational studies, precluding the establishment of a causal relationship between frailty and prolonged survival in GC. Consequently, clinical investigations may be warranted to assess the efficacy of interventions targeting frailty in enhancing the survival of GC patients.

Based on the results of the meta-analysis, frailty may contribute to unfavorable long-term survival outcomes in GC patients. Further research is warranted to identify the most effective screening tool for detecting frailty in cancer patients, and to investigate whether interventions aimed at addressing frailty are associated with improved long-term survival rates among GC patients.

## Data availability statement

The original contributions presented in the study are included in the article/supplementary material. Further inquiries can be directed to the corresponding author.

## Author contributions

HL designed the study. HL and AH performed literature search, study selection, study quality evaluation, and data collection. HL and AH conducted statistical analyses and interpreted the results. HL drafted the manuscript. HL and AH revised the manuscript and approved the submission. All authors contributed to the article and approved the submitted version.
